# Supplementary figures and images for: Behavioral Phenotyping of WAG/Rij Rat Model of Absence Epilepsy: The Link to Anxiety and Sex Factors
Source: Biomedicines. 2025 Aug 26;13(9):2075. doi: 10.3390/biomedicines13092075 (PMC12467176; doi:10.3390/biomedicines13092075)

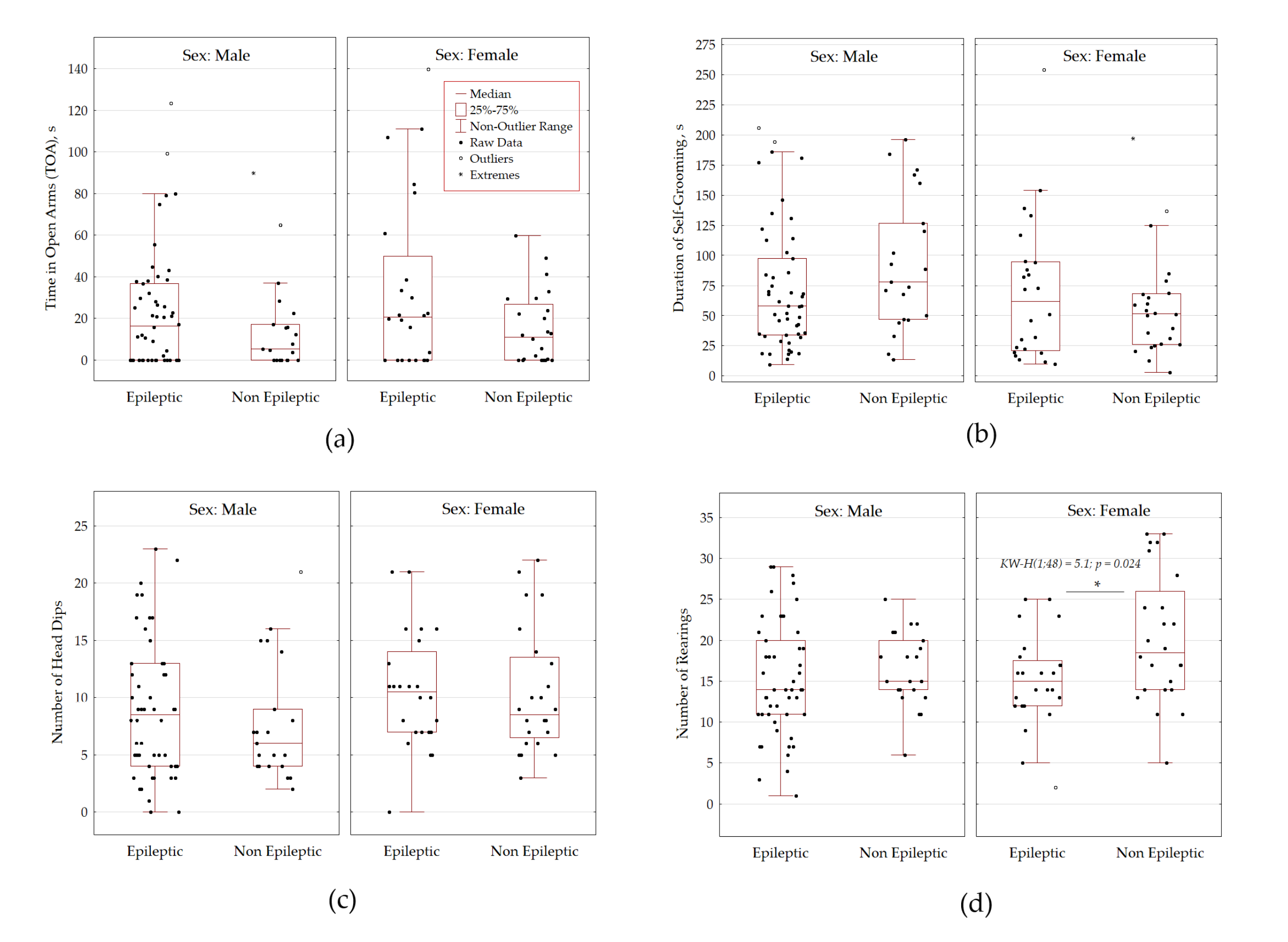

Supplement: Supplementary file 1 [file biomedicines-13-02075-s001.zip › biomedicines-3820578-Figure S1.png]
